# Herd-level based seroprevalence and associated factors for *Toxoplasma gondii* in cows in the state of Paraíba, Northeastern Brazil

**DOI:** 10.1590/S1984-29612023025

**Published:** 2023-05-05

**Authors:** Amanda Rafaela Alves Maia, Roberto Alves Bezerra, Samara Santos Silva, Felipe Boniedj Ventura Álvares, Carolina de Sousa Américo Batista Santos, Clebert José Alves, Inácio José Clementino, Thais Ferreira Feitosa, Vinícius Longo Ribeiro Vilela, Sérgio Santos de Azevedo

**Affiliations:** 1 Unidade Acadêmica de Medicina Veterinária, Centro de Saúde e Tecnologia Rural, Universidade Federal de Campina Grande - UFCG, Patos, PB, Brasil; 2 Laboratório de Imunologia e Doenças Infectocontagiosas, Instituto Federal da Paraíba - IFPB, Sousa, PB, Brasil; 3 Departamento de Medicina Veterinária, Centro de Ciências Agrárias, Universidade Federal da Paraíba - UFPB, Areia, PB, Brasil

**Keywords:** Zoonosis, toxoplasmose, two-stage sample survey, cattle herd, *T. gondii* infection, Zoonose, toxoplasmosis, amostragem em dois estágios, rebanho bovino, infecção por *T. gondii*

## Abstract

We aimed to determine the herd and animal levels seroprevalence and associated factors for *Toxoplasma gondii* infections in cattle from the state of Paraíba, Northeastern Brazil. Herds (n = 434) and cows aged ≥ 24 months (n = 1,895) were randomly selected, and serum samples were tested with the immunofluorescence antibody test (IFAT) using as cutoff of 64. Of the 434 farms investigated, 197 had at least one seropositive cow (prevalence of 49.0%; 95% CI = 44.3%-53.8%), and the prevalence at animal level was 18.0% (95% CI = 5.3%-21.1%). The antibody titers ranged from 64 to 1024, with the most frequent titers being 64 (10.8%) and 128 (3.7%). The risk factors identified were property located in Sertão region (odds ratio [OR] = 3.07), property located in Agreste/Zona da Mata regions (OR = 2.00), animal purchasing (OR = 2.68), herd size of 34-111 animals (OR = 2.91) and herd size > 111 animals (OR = 6.97). The results suggest the wide distribution of *T. gondii* infections in cattle throughout the state of Paraíba, and the risk factors identified are not possible to correct.

## Resumo

Objetivou-se determinar a soroprevalência do rebanhos e de animais e os fatores associados à infecção por *Toxoplasma gondii* em bovinos do estado da Paraíba, Nordeste do Brasil. Rebanhos (n = 434) e vacas com idade ≥ 24 meses (n = 1.895) foram selecionados aleatoriamente, e as amostras de soro foram testadas com o teste de imunofluorescência (RIFI), usando-se como ponto de corte 64. Das 434 fazendas investigadas, 197 tiveram pelo menos uma vaca soropositiva (prevalência de 49%; IC 95% = 44,3%-53,8%), e a prevalência em nível de animais foi de 18% (IC 95% = 5,3%-21,1%). Os títulos de anticorpos variaram de 64 a 1024, sendo os títulos mais frequentes 64 (10,8%) e 128 (3,7%). Os fatores de risco identificados foram na propriedade localizada na região do Sertão ("odds ratio" [OR] = 3,07), na propriedade localizada na região do Agreste/Zona da Mata (OR = 2,00), na compra de animais (OR = 2,68), no tamanho do rebanho de 34-111 animais (OR = 2,91) e no tamanho do rebanho > 111 animais (OR = 6,97). Os resultados sugerem a ampla distribuição de infecções por *T. gondii* em bovinos em todo o estado da Paraíba, e os fatores de risco identificados não são passíveis de correção.

## Introduction

Toxoplasmosis is a parasitic zoonosis, caused by the obligate intracellular protozoan *Toxoplasma gondii*, which is capable of infecting most homeothermic species, including humans, by forming tissue cysts. The infection is generally asymptomatic and does not cause clinical disease in several animal species, but in some, it causes acute life-threatening illness (Smith et al., 2021). Humans can become infected mainly by eating undercooked, contaminated meat (especially pork, lamb, and venison) or shellfish (like oysters, clams, and mussels); accidentally ingesting undercooked, contaminated meat or shellfish after handling it and not washing hands thoroughly; eating food that was contaminated by knives, utensils, cutting boards or other foods that had contact with raw, contaminated meat or shellfish; and drinking unpasteurized goat’s milk (Dubey, 2010; CDC, 2023).

Felids are important in the life cycle of *T. gondii* because they are definitive hosts and, therefore, the only ones that can contaminate the environment with oocysts, which after sporulating, become infective, spreading through soil, water and vegetation, and because they are resistant they can survive in humid environments for several months (Dubey et al., 2020). The risk factors associated with *T. gondii* infection in animals is similar to that in humans, and depends on the type of geographical region, the sanitary conditions and management of the farms, and the animal's diet (origin of the water and food offered). The most common source of infection in animals is through the ingestion of sporulated oocysts, either through food or water. Carnivores are infected most commonly by oral route (more than 80% of cases), through consumption of raw or undercooked meat containing tissue cysts, or vegetables and fruits contaminated with oocysts shed by felids (Jones & Dubey, 2012; Hill & Dubey, 2013).

In veterinary medicine, toxoplasmosis represents a serious problem and is considered a major cause of reproductive losses in sheep, goats and pigs (Ferra et al., 2020). Although it is considered a poor host for *T. gondii*, natural and experimental infections in cattle have been reported (Dubey, 2010; Costa et al., 2011). Vertical transmission should also be considered, since in experimental infection, inoculated pregnant females aborted or gave birth to congenitally infected offspring (without apparent clinical signs), favoring the maintenance of the agent in the herd (Wiengcharoen et al., 2011).

The Brazilian cattle herd has reached 218.2 million heads and the data from the Brazilian Institute of Geography and Statistics report that by the third quarter of 2021, 20,614,976 million cattle were slaughtered, taking into account establishments under federal, state or municipal health inspection, in which 40,420 of these cattle correspond to the state of Paraíba (IBGE, 2021), which shows the importance of this food. Due to the isolation of this protozoan in bovine tissues, the importance of this infection in the epidemiological chain of toxoplasmosis cannot be neglected (Gomes et al., 2020).

Studies on bovine toxoplasmosis are of utmost importance due to the zoonotic potential. Therefore, the objective of this study was to determine the seroprevalence of toxoplasmosis in cows in the state of Paraíba, Northeastern Brazil, using a planned sampling targeting herds and animals, as well as to identify the risk factors associated with herd-level seroprevalence.

## Material and Methods

### Characterization of the study area

**Figure 1:**
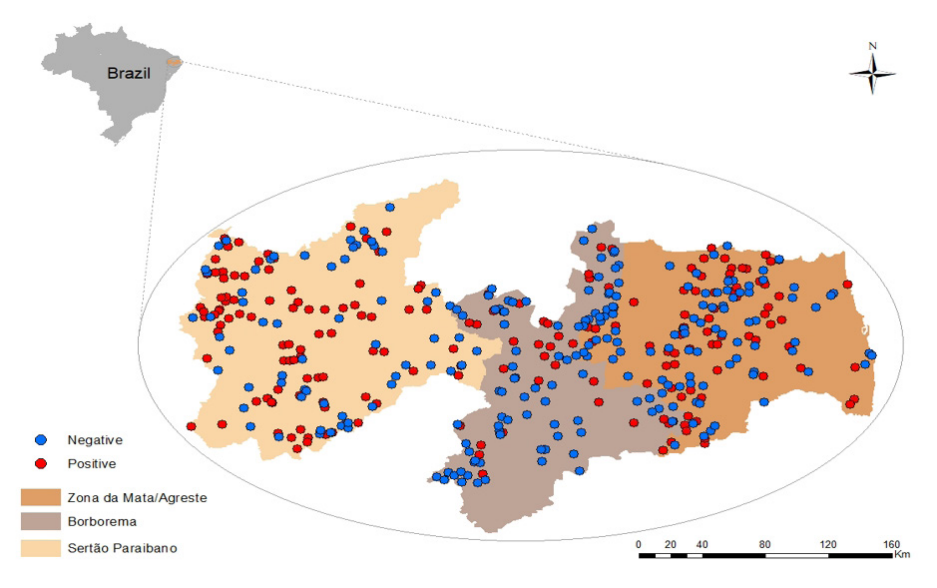
Division of Paraíba state into three sample strata (Sertão, Borborema, and Agreste/Zona da Mata) and distribution of positive and negative cattle herds for *T. gondii* infections.

The study was carried out in Paraíba state, Northeast region of Brazil. The state was stratified according to the operational capacity of the Animal Defense Service of the State of Paraíba (SEDAP) based on the areas of operation of its regional offices to ensure that the agency could perform the field work. It was divided into three sampling groups: sampling stratum 1 (mesoregion of Sertão), sampling stratum 2 (mesoregion of Borborema) and sampling stratum 3 (mesoregions of Zona da Mata and Agreste) (Figure 1).

### Sampling

The serum samples used in this study were obtained from a bovine brucellosis study in the state of Paraíba, conducted by the National Program for the Control and Eradication of Brucellosis and Tuberculosis (Clementino et al., 2016), and the sampling design was adjusted for bovine toxoplasmosis. For each sampling stratum, the prevalence of herds and the prevalence of seropositive animals were estimated by two-stage sample survey. In the first stage, a predetermined number of herds (primary sampling units) were randomly selected; in the second stage, a predetermined number of cows aged ≥ 24 months were randomly selected (secondary sampling units).

The selection of the primary sampling units was random (random draw) and was based on the SEDAP farm registers. The number of herds selected per sampling stratum was determined using the formula for simple random sampling (Thrusfield & Christley, 2018). The parameters adopted for the calculation were as follows: confidence level of 95%, estimated prevalence of 71% (Santos et al., 2009) and 5% error. In addition, the operational and financial capacity of SEDAP was taken into account in determining the sample size.

For the secondary units, the minimum number of animals to be examined within each herd was estimated in order to allow their classification as positive. For this, the concept of aggregate sensitivity and specificity was used (Dohoo et al., 2003). For the calculations, we adopted 100% for the sensitivity and specificity of the serological test (Sunanta et al., 2009), and 71% for the estimated intra-herd prevalence (Santos et al., 2009). Herdacc version 3 software (Jordan, 1995) was used during this process and the sample size was selected so that the herd sensitivity and specificity values were ≥ 90%. Therefore, 10 animals were sampled in herds with up to 99 cows older than 24 months; 15 animals were sampled in herds with 100 or more cows older than 24 months; and all animals were sampled in those with up to 10 cows older than 24 months. The selection of cows within herds was systematic.

### Field activites

The field activities included blood collection, application of an epidemiological questionnaire, and sending the samples to the laboratory. SEDAP veterinarians and agricultural and livestock technicians were involved in the field work. Blood samples (10 mL volume) were collected from September 2012 to January 2013, from cows aged ≥ 24 months by puncture of the jugular vein with disposable needle and vacuum tube with 15 mL capacity (without anticoagulant).

### Immunofluorescence antibody test (IFAT)

Serological analyses were performed in the Laboratory of Immunology and Infectious Diseases (LIID) of the Adílio Santos Azevedo Veterinary Hospital (ASA) of the Federal Institute of Paraíba (IFPB), Sousa-PB. For detection of anti-*T. gondii* antibodies, serum samples were submitted to IFAT, considering the dilution of 1:64 as cutoff point (Santos et al., 2009). Tachyzoites of *T. gondii* strain ME-49, maintained in mice, were used as antigens. Positive and negative control bovine sera were kindly provided by Prof. Dr. Rinaldo Aparecido Mota, Universidade Federal Rural de Pernambuco (UFRPE), Brazil. The conjugate used in the reactions was anti-bovine IgG (whole molecule with FITC, produced in rabbits, Sigma/F-7887), at a dilution of 1:400 in PBS 7.2 solution containing 10% Evan’s Blue. Reactions were considered positive when tachyzoites showed full peripheral fluorescence. Reactive serum samples were titrated in serial dilutions until the highest positive dilution was obtained.

### Calculation of prevalence

The target condition was a seropositive animal in an infected herd. The herd-level case definition was based on population size (cows aged ≥ 24 months), number of females sampled, an apparent intra-herd prevalence of 71% (Santos et al., 2009), and the sensitivity and specificity of serology, with the goal of obtaining herd sensitivity and specificity ≥90%. After several simulations with the Herdacc software, a herd was deemed positive for toxoplasmosis if it had at least one seropositive animal.

EpiInfo 6.04 software was used to calculate apparent prevalences and their confidence intervals (Dean et al., 1996). Stratified random sampling was used to calculate herd-level prevalence in Paraíba state (Thrusfield & Christley, 2018). Required parameters were (a) herd condition (positive or negative), (b) sampling stratum to which the herd belonged, and (c) statistical weight. The statistical weight was determined by applying the following formula (Dean et al., 1996):


$$Weight=\frac{\text{number of herds in the stratum}}{\text{number of herds sampled in the stratum}}$$ (1)

The calculation of herd-level prevalence per sampling stratum employed a simple random sample design using the following parameters: (a) number of positive herds and (b) number of herds sampled in the stratum. The sampling design for the calculation of animal-level prevalence in Paraíba state used a two-stage stratified cluster sampling and a two-stage cluster sampling in each stratum (Thrusfield & Christley, 2018), and each herd was considered a cluster. The following parameters were used: (a) animal status (seropositive or seronegative), (b) sample stratum to which the animal belonged, (c) herd code (to identify each cluster), and (d) statistical weight. The statistical weight was calculated with the following formula (Dean et al., 1996):


$$Weight=\frac{\text{cows}\geq \text{24 months in the stratum}}{\text{cows}\geq \text{24 months in the sampled herds}}\times \frac{\text{cows}\geq \text{24 months in the herd}}{\text{cows}\geq \text{24 months sampled in the herd}}$$ (2)

### Risk fator analysis

The variables obtained with the epidemiological questionnaires were organized for presentation in increasing or decreasing order as to the scale of risk. The variable herd size was categorized according to quartiles. The lowest risk category was considered the basis of comparison for the other categories. An initial exploratory data analysis (univariable) was performed by the chi-square test or Fisher's exact test, and variables with P ≤ 0.2 were selected for logistic regression analysis (Hosmer & Lemeshow, 2000). The final model fit was verified with the Hosmer and Lemeshow test and collinearity between the independent variables was checked by correlation analysis; for variables with strong collinearity (correlation coeficiente > 0.9), one of the two variables was excluded from the multiple analysis according to biological plausibility (Dohoo et al., 2003). The confounding variables was assessed by monitoring changes in model parameters when adding new variables. If substantial changes (i.e. greater than 20%) were observed in the regression coefficients, this was considered an indication of confounding. The significance level in final model was 5%, and the calculations were performed in SPSS software version 20.0.

## Results

The census data and the sample studied in each sampling stratum are presented in Table 1. In total, 1,895 animals were sampled in 434 farms. Herd and animal level prevalences are presented in Table 2; in addition, the geographic distribution of positive and negative herds is shown in Figure 1. Of the 434 farms investigated, 197 had at least one seropositive cow (prevalence of 49.0%; 95% CI = 44.3%-53.8%). In Sertão, the prevalence was 60.3% (95% CI = 52.1%-67.9%), 26.5% (95% CI = 19.7%-34.6%) in Borborema and 48.3% (95% CI = 40.2%-56.5%) in Agreste/Zona da Mata. The prevalence at animal level was 18.0% (95% CI = 5.3%-21.1%) in Paraíba state, 18.5% (95% CI = 15.2%-22.5%) in Sertão, 15.7% (95% CI = 7.6%-29.5%) in Borborema and 18.3% (95% CI = 14%-23.4%) in Agreste/Zona da Mata. The titers of anti-*T. gondii* antibodies ranged from 64 to 1024, with the most frequent titers being 64 (10.8%) and 128 (3.7%).

**Table 1:** Census data on the cattle population in Paraíba state, Northeastern Brazil, according to sampling stratum.

Sampling stratum	No. of herds		No. of cows ≥ 24 months of age	
	Total	Sampled	Total	Sampled
Sertão	24,356	151	288,764	854
Borborema	11,603	136	83,428	356
Agreste/Zona da Mata	18,398	147	192,320	685
State of Paraíba	54,357	434	564,512	1,895

**Table 2:** Herd and animal levels prevalence of *T. gondii* infections in cattle from the state of Paraíba, Northeastern Brazil, according to sampling stratum.

Sampling stratum	Herd-level				Animal-level			
	Tested	Positive	Prevalence (%)	95% CI	Tested	Positive	Prevalence (%)	95% CI
Sertão	151	91	60.3	52.1-67.9	854	158	18.5	15.2-22.5
Borborema	136	36	26.5	19.7-34.6	356	47	15.7	7.6-29.5
Agreste/Zona da Mata	147	70	48.3	40.2-56.5	685	115	18.3	14-23.4
State of Paraíba	434	197	49.0	44.3-53.8	1,895	320	18.0	5.3-21.1

The results of the univariable analysis for the risk factors are presented in Table 3. The variables selected (P ≤ 0.2) for the multiple analysis were region, management system, herd size, presence of wild animals, occurrence of abortions, animal purchasing, sharing of pastures, sharing of water sources, use of maternity pens, veterinary assistance and type of property. In the final logistic regression model (Table 4), the risk factors identified were property located in Sertão (odds ratio [OR] = 3.07), property located in Agreste/Zona da Mata (OR = 2.00), animal purchasing (OR = 2.68), herd size of 34-111 animals (OR = 2.91) and herd size > 111 animals (OR = 6.97). The final model had a good fit (Hosmer and Lemeshow test: chi-square = 1.498; P = 0.983).

**Table 3:** Univariable analysis for factors associated with the herd-level prevalence of *T. gondii* infections in cattle, in the state of Paraiba, Northeastern Brazil.

Variables	Categories	No. of herds sampled	No. of positive herds (%)	*P*
Region	Sertão	151	91 (60.3)	
	Borborema	136	36 (26.5)	
	Agreste/Zona da Mata	147	71 (48.3)	< 0.001*
				
				
Type of production	Beef	59	21 (35.6)	
	Dairy	123	57 (46.3)	
	Mixed	252	120 (47.6)	0.244
				
Management system	Intensive	29	7 (24.1)	
	Semi-intensive	257	111 (43.2)	
	Extensive	148	80 (54.1)	0.006*
				
Artificial insemination	Not use	432	197 (45.6)	
	Use only insemination	2	1 (50.0)	0.705
				
Herd size	Up to 33 animals	218	58 (26.6)	
	34 - 111 animals	108	59 (54.6)	
	> 111 animals	108	81 (75.0)	< 0.001*
				
Presence of cats	No	255	121 (47.5)	
	Yes	179	77 (43.0)	0.208
				
Presence of wild animals	No	264	126 (47.7)	
	Yes	170	72 (42.4)	0.159*
				
Occurrence of abortions	No	398	178 (44.7)	
	Yes	36	20 (55.6)	0.141*
				
Animals purchasing	No	391	171 (43.7)	
	Yes	43	27 (62.8)	0.013*
				
Animal saling	No	317	142 (44.8)	
	Yes	117	56 (47.9)	0.322
				
Local of animal slaughter	Not slaughter	196	86 (43.9)	
	In slaughterhouses	154	74 (48.1)	
	In establishment not inspection	81	35 (43.2)	
	In own farm	3	3 (100.0)	0.224
				
Rental of pastures	No	341	154 (42.5)	
	Yes	93	44 (47.3)	0.400
				
Sharing of pastures	No	370	165 (44.6)	
	Yes	64	33 (51.6)	0.185*
				
Sharing of water sources	No	371	163 (43.9)	
	Yes	63	35 (55.6)	0.058*
				
Presence of flooded pastures	No	288	134 (46.5)	
	Yes	146	64 (43.8)	0.334
				
Use of maternity pens	No	322	138 (42.9)	
	Yes	112	60 (53.6)	0.032*
				
Raw milk consumption	No	366	166 (45.4)	
	Yes	68	32 (47.1)	0.449
				
Veterinary assistance	No	364	156 (42.9)	
	Yes	70	42 (60.0)	0.006*
				
Type of property	Rural	218	58 (26.6)	
	Indian vilage	108	59 (54.6)	
	Rural settlement	108	81 (75.0)	< 0.001^*^
				

* Variables selected and used in the multiple analysis (*P* ≤ 0.2).

**Table 4:** Logistic regression final model with factors associated with the herd-level prevalence of *T. gondii* infections in cattle from the state of Paraíba, Northeastern Brazil.

Associated factor	Logistic regression coefficient	Standard error	Wald	Odds ratio	(95% CI)	P-value
Property located in Sertão	1.122	0.278	16.222	3.07	(1.78-5.30)	< 0.001
Property located in Agreste/Zona da Mata	0.692	0.276	6.276	2.00	(1.16-3.44)	0.012
Animal purchasing	0.984	0.372	6.995	2.68	(1.29-5.55)	0.008
Herd size of 34 - 111 animais	1.067	0.257	17.286	2.91	(1.76-4.81)	< 0.001
Herd size > 111 animais	1.942	0.279	48.429	6.97	(4.04-12.05)	< 0.001

## Discussion

In this study, a comprehensive epidemiological survey for toxoplasmosis was performed with planned sampling to determine the seroprevalence at herd and animal levels in cattle in Paraíba state. Only samples from cows aged ≥ 24 months were used because there was a pre-existing serum bank. In addition, females are animals that remain longer in the herd and can provide a more accurate profile of infection, and are animals that contribute to the maintenance of the agent circulating in herds due to the possibility of vertical transmission.

Despite we used serum samples collected between September 2012 to January 2013, a serum bank is ‘a planned catalogued collection of serum forming a random sample that is as representative as possible of a population and that is stored to preserve its immunological and biochemical characteristics’ (Thrusfield & Christley, 2018).

This survey was carried out in all regions of the state of Paraíba, with herd-level prevalence of 49% (95% CI = 44.3%-53.8%) and positive herds covering all mesoregions. This suggests broad capacity of dissemination and adaptability of *T. gondii* (Tenter et al., 2000) and hypothesizes environmental contamination by the parasite. The occurrence of infection due to *T. gondii* in the Brazilian cattle herd is variable with frequencies from 1% (Gondim et al., 1999) to 89.1% (Santin et al., 2017), however studies should be compared with caution due to the use of different diagnostic techniques with different cutoff points (Gomes et al., 2020).

In Brazil, studies on the frequency of anti- *T. gondii* antibodies in cattle have been based mainly on IFAT (Dubey et al., 2012), and in a review (Gomes et al., 2020) of the 35 studies conducted in Brazil 24 (68.5%) used IFAT as diagnostic method, among which 22 (91.6%) established 64 as the cutoff point, as in this study. The most frequent antibody titer obtained in this study was 64, corresponding to 10.8%, followed by 128 (3.7%), results similar to those obtained by Gomes et al. (2020). Dubey & Thulliez (1993) support that because of resistance to toxoplasmosis, antibody titers lower than 1,024 are indicative of chronic infection, suggesting the presence of tissue cysts.

Although the role of cattle in the transmission of the parasite to humans is not completely known, beef is often eaten undercooked and may pose a risk to the population (Gomes et al., 2020). Cattle can be readily infected with *T. gondii*, but are considered poor hosts because they develop a more effective immune response to *T. gondii* infection than other animals (Esteban-Redondo & Innes, 1997). However, *T. gondii* has been isolated from bovine tissues and unpasteurized milk (Dubey, 1986), indicating that consumption of meat and milk can be source of *T. gondii* transmission. The ingestion of beef of questionable provenance increases the risk of *T. gondii* infection to the consumer (Millar et al., 2008), and due to the informality of slaughterhouses and food markets in Brazil, especially in Paraíba where there is no federal inspection for cattle (Maia et al., 2017), the clandestine slaughter is a worrisome factor.

*T. gondii* infection is often more common in areas with warm, humid climates and lower altitudes (Dubey, 2010), characteristics of the entire state of Paraíba. The average temperatures of the Northeast region are between 26º and 28 ºC, with low annual variability, and the state of Paraíba, due to its location within the Equatorial belt, is subjected to the incidence of high solar radiation with a large number of hours of insolation. Such condition determines a warm climate and average annual temperature of 26 ºC, also with low intra-annual variation (AESA, 2016). In this study, the Sertão and Agreste/Zona da Mata mesoregions presented association with *T. gondii* prevalence. It is known that *T. gondii* infection in herbivores is more prevalent in humid areas, which favor sporulation conditions and maintenance of oocyst viability in vegetation (Dubey, 2010). Brejo Paraibano, a microregion that belongs to Agreste, has characteristics with the presence of Atlantic Forest fragments, high rainfall, and a wide variety of fauna, which may favor the viability of oocysts in this region.

Sertão is an area bordering the states of Rio Grande do Norte, Ceará and Pernambuco, where there is an intense trade of animals without the knowledge of their sanitary condition, which may justify this region as risk factor. It is noteworthy that, despite the climatic conditions of the Sertão being different from the Agreste, which presents favorable abiotic factors for *T. gondii*, the family farm production in the Sertão, with low technification level of the properties, and without the support of important general sanitary measures for the control of infectious diseases, as quarantine of animals coming from other regions (Clementino et al., 2015) are possible factors that may be generating this risk association. In addition, primary clusters of positive herds for important cattle infectious diseases (such as Bovine Viral Diarrhea Virus, Bovine Herpesvirus type 1 and Vesicular Stomatitis) covering the Sertão region were detected in previous surveys (Bezerra et al., 2018; Fernandes et al., 2018).

Animal purchasing was also identified as associate with *T. gondii* prevalence. This variable is a classical risk fator for infectious diseases and is related to acquisition of animals without previous testing for diseases. In fact, the serological testing of cattle for toxoplasmosis prior to introducting into the herd is not a common procedure anywhere, which facilitates the introduction of infected animals. The producer often purchases animals without knowing the origin, and some diseases are not previously diagnosed due to the high cost and difficult accessibility of diagnostic tests. In Brazil, this variable has been identified as risk factor for several bovine diseases, such as toxoplasmosis (Gomes et al., 2021) in the Brazilian cerrado, leptospirosis (Hashimoto et al., 2012) and neosporosis (Gindri et al., 2018) in Rio Grande do Sul, as well as in Northeastern Brazil for brucellosis (Silva et al., 2009), neosporosis (Silva et al., 2008), bovine viral diarrhea (Fernandes et al., 2016) and cysticercosis (Maia et al., 2017). Herd size ≥ 34 animals was associated with herd-level prevalence, indicating that the larger the herd size the greater the odds of herd positivity. In fact, properties with a great number of animals tended to introduce new animals more often, a practice that, without sanitary precautions, may predispose the introduction of toxoplasmosis into the herd.

In conclusion, the results found here suggest the wide distribution of *T. gondii* infections in cattle throughout the state of Paraíba, Northeastern Brazil, and the risk factors identified are not possible to correct.
